# Cardiovascular magnetic resonance native T1 mapping in Anderson-Fabry disease: a systematic review and meta-analysis

**DOI:** 10.1186/s12968-022-00859-z

**Published:** 2022-05-23

**Authors:** Andrea Ponsiglione, Michele Gambardella, Roberta Green, Valeria Cantoni, Carmela Nappi, Raffaele Ascione, Marco De Giorgi, Renato Cuocolo, Antonio Pisani, Mario Petretta, Alberto Cuocolo, Massimo Imbriaco

**Affiliations:** 1 Department of Advanced Biomedical Sciences, University of Naples Federico II, Via Pansini 5, 80131 Naples, Italy; 2 Department of Clinical Medicine and Surgery, University of Naples Federico II, Naples, Italy; 3 Department of Public Health, University of Naples Federico II, Naples, Italy; 4 Interdepartmental Research Center on Management and Innovation in Healthcare (CIRMIS), University of Naples Federico II, Naples, Italy; 5 IRCCS Synlab SDN Spa, Naples, Italy

**Keywords:** Anderson-Fabry disease, CMR, T1 mapping, Systematic review, Meta-analysis

## Abstract

**Background:**

T1 mapping is an established cardiovascular magnetic resonance (CMR) technique that can characterize myocardial tissue. We aimed to determine the weighted mean native T1 values of Anderson-Fabry disease (AFD) patients and the standardized mean differences (SMD) as compared to healthy control subjects.

**Methods:**

A comprehensive literature search of the PubMed, Scopus and Web of Science databases was conducted according to the PRISMA statement to retrieve original studies reporting myocardial native T1 values in AFD patients and healthy controls. A random effects model was used to calculate SMD, and meta-regression analysis was conducted to explore heterogeneity sources. Subgroup analysis was also performed according to scanner field strength and sequence type.

**Results:**

From a total of 151 items, 14 articles were included in the final analysis accounting for a total population of 982 subjects. Overall, the weighted mean native T1 values was 984 ± 47 ms in AFD patients and 1016 ± 26 ms in controls (*P* < 0.0001) with a pooled SMD of − 2.38. In AFD patients there was an inverse correlation between native T1 values and male gender (*P* = 0.002) and left ventricular hypertrophy (LVH) (*P* < 0.001). Subgroup analyses confirmed lower T1 values in AFD patients compared to controls with a pooled SMD of −  2.54,  −  2.28, −   2.46 for studies performed on 1.5T with modified Look-Locker inversion recovery (MOLLI), shortened MOLLI and saturation-recovery single-shot acquisition, respectively and of −  2.41 for studies conducted on 3T.

**Conclusions:**

Our findings confirm a reduction of native T1 values in AFD patients compared to healthy controls and point out that the degree of T1 shortening in AFD is influenced by gender and LVH. Although T1 mapping is useful in proving cardiac involvement in AFD patients, there is need to standardize shreshold values according to imaging equipment and protocols.

## Background

Anderson-Fabry disease (AFD) is a rare multisystem X-linked lysosomal storage disorder caused by α-galactosidase A enzyme deficiency, resulting in progressive intracellular accumulation of glycosphingolipids in endothelial and smooth muscle cells [1, 2]. Clinical features of classic AFD phenotype consist in skin disorders, corneal alterations, cerebrovascular complications, kidney failure, and cardiovascular disease, which represents a major cause of morbidity and mortality [3, 4]. Long-term cardiac involvement in AFD results in left ventricular (LV) hypertrophy (LVH) and myocardial fibrosis, inducing several complications, mainly arrhythmias, valvular dysfunction, and coronary artery disease [5]. Cardiovascular magnetic resonance (CMR) imaging is the leading non-invasive imaging modality for cardiac involvement in AFD, as it allows a comprehensive assessment of cardiac anatomy, regional and global ventricular function, and tissue characterization [6, 7]. Detection and quantification of myocardial replacement fibrosis by late gadolinium enhancement (LGE) is a crucial prognostic feature in clinical risk stratification of AFD patients, relating to hard cardiovascular outcomes [8]. T1 mapping is an established and reproducible emerging CMR technique for characterization of myocardial tissue by identification of myocardial edema, intra-myocyte lipids accumulation, and extracellular volume expansion (proteins or iron deposition) [9, 10]. In particular, native T1 mapping estimates the intrinsic longitudinal relaxation time of myocardial tissue without the need for a contrast agent. Several techniques are available for generating T1 maps, each of them characterized by specific advantages and disadvantages related to spatial resolution, acquisition time and accuracy [10].

Cardiac glycosphingolipids overload in early stages of AFD induces reduction of myocardial native T1 values and its identification may precede the development of LVH and myocardial dysfunction [11]. However, T1 values are influenced by patients’ demographic characteristics as well as by the magnetic field strength and acquisition protocol used. The aim of this systematic review and meta-analysis was to summarize the weighted mean native T1 values in AFD patients and to determine which factors influence the degree of T1 shortening compared to healthy subjects according to patient’s clinical characteristics and different imaging systems and protocols.

## Methods

This meta-analysis followed the PRISMA (Preferred Reporting Items for Systematic Reviews and Meta- Analyses) statement (supplementary material for PRISMA Checklist) [12]. The review protocol is registered with PROSPERO (CRD42021285223).

### Search strategy

An English literature search was performed using the PubMed, Scopus and Web of Science databases to identify articles published up to April 2021. The following search terms and their variations were used: “Fabry” and “T1” and “mapping” and “cardiac magnetic resonance imaging”. The detailed search string is shown in the supplemental material (Appendix).

### Study selection

The title and abstract of potentially relevant studies were screened for appropriateness before retrieval of the full article by two reviewers (R.G. and V.C.), and disagreements were resolved by consensus. The full-published reports of the selected abstracts were retrieved, and the same reviewers independently performed a second-step selection based on the study eligibility, and disagreements were resolved by consensus. In addition, the reference lists of the retrieved articles were manually reviewed to find potentially eligible studies missed in the primary search.

### Study eligibility and data extraction

Each study was initially identified considering journal, authors, and year of publication. A study was considered eligible if all the following criteria were met: (1) a cohort, case–control, or cross-sectional design; (2) native T1 mapping assessed with CMR were provided in adult patients with AFD and control subjects; (3) modified Look-Locker inversion recovery (MOLLI) T1 mapping from 1.5T or 3T CMR scanners, shortened MOLLI (ShMOLLI), and saturation-recovery single-shot acquisition (SASHA) pulse sequence with a balanced steady-state free precession readout was allowed. In case of studies reporting data according to different AFD population categories with and without LVH, we considered them separately. Reviews, editorials, abstracts, animal studies, conference presentations, studies not focused on the topic of interest or published in languages other than English were excluded. Relevant data regarding characteristics for both AFD patients and control subjects, such as study population, age, gender, LVH, LV ejection fraction (LVEF) and native T1 values were retrieved. CMR imaging acquisition related information, such as field strength, vendor, sequence type, sequence parameters and region of interest (ROI) sampling were also collected.

### Quality assessment

Methodological quality of included studies was performed independently by the two reviewers (R.G. and V.C.) with the Newcastle–Ottawa quality assessment scale (NOS) [13]. This scale evaluated the study quality on three domains: selection and definition of included populations (0–4 points); comparability of the controls (0–2 points); and ascertainment of the outcome (0–3 points).

### Statistical analysis

Continuous variables were expressed as mean ± standard deviation and categorical data as percentages. T1 values for AFD patients and for control subjects were combined into a random effects model to determine the standardized mean difference (SMD) and 95% confidence interval (CI) weighted by the inverse of variance. Heterogeneity of the included studies was examined by using the I-squared (I^2^) statistic, to reflect the percentage of total variation across studies, assigning adjectives of low, moderate, and high to I^2^ values of 25%, 50%, and 75%. According to the Cochrane handbook, I^2^ > 50% reflects a substantial heterogeneity. When statistical heterogeneity was substantial, meta-regressions were performed for each confounder to examine possible study factors associated with heterogeneity. Included covariates were at least gender, age, LV ejection fraction, and LVH. Beta coefficients were derived using least-mean square fitting method. Publication bias was assessed by inspection of the funnel plots with the Egger regression asymmetry test. Subgroup analysis was also performed dividing the studies according to 1.5T and 3T scanners, and MOLLI, ShMOLLI and SASHA acquisitions. Meta-regression and publication bias analyses were performed in each population with at least 10 published studies, as stated by the PRISMA guideline. All analysis were performed using Stata (version 15.1, StataCorp, College Station, Texas, USA). Two-sided *P* values < 0.05 were considered statistically significant.

## Results

### Study selection

**Fig. 1:**
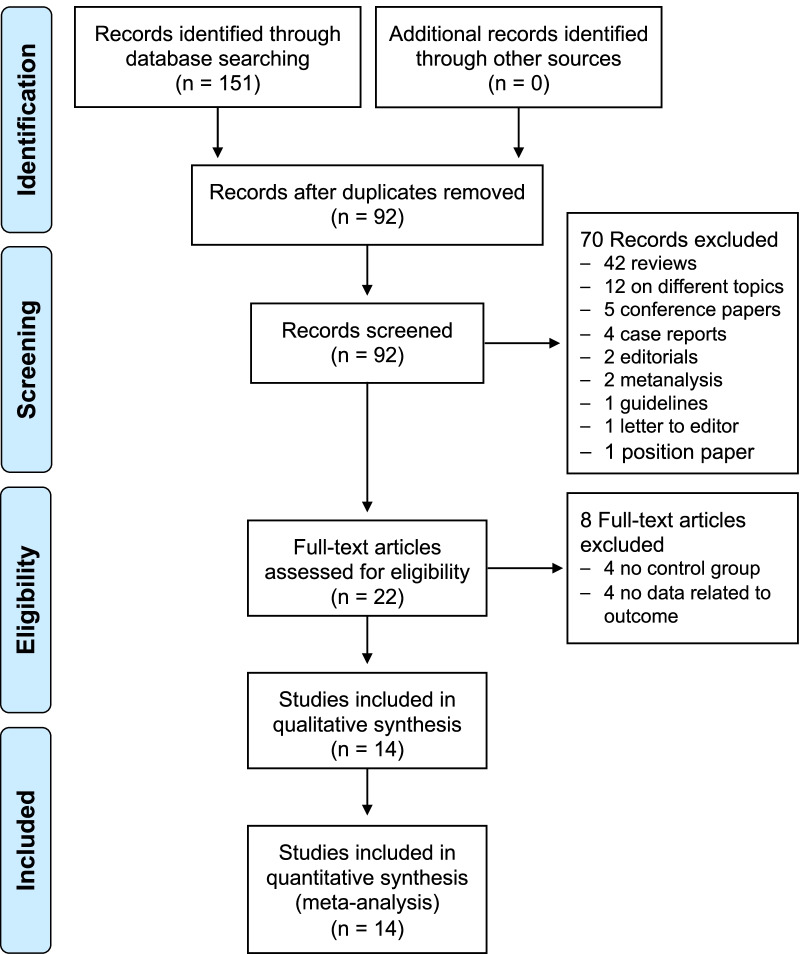
PRISMA flowchart illustrating the study selection process

The complete literature search is presented in Fig. 1. The initial search identified 151 potentially eligible articles. Among these, 59 were identified as duplicates and removed, leaving 92 studies. The reviewers, after the evaluation of the titles and abstracts of these latter studies removed 70 citations. Then, each investigator blindly reviewed the full text of the remaining 22 articles, and 8 articles were excluded. The remaining 14 studies, including a total of 982 subjects (477 AFD patients and 505 control subjects), were the basis of the present meta-analysis [14–27].

### Study characteristics

Characteristics and imaging data of the included studies are detailed in the Table 1. Eleven studies were conducted prospectively [14, 15, 17–21, 24–27] and 3 retrospectively [16, 22, 23]. Most of the investigations were based on single center experiences [14–17, 19–27]. Ten studies used 1.5T scanner [15, 16, 18, 20, 22–27] and 4 studies 3T scanner [14, 17, 19, 21]. Demographic characteristics of the subjects included in the meta-analysis are reported in Table 2. Sample size ranged from 6 to 72 for AFD patients and from 7 to 76 for control subjects, with a mean age of 45 ± 22 years in AFD patients and of 44 ± 21 years in control subjects. The proportion of men ranged from 0 to 95% for AFD patients and from 0 to 64% for control subjects.

**Table 1:** Characteristics and Imaging Data of the Included Studies

Reference	Study design	Study type	Field strength	Sequence	ROI sampling	Quality score	LVH (%)		LVEF (%)		Native T1 (ms)	
							AFD patients	Control subjects	AFD patients	Control subjects	AFD patients	Control subjects
[15]	Prospective	Single center	1.5T	MOLLI	Mean basal-mid SAx	2, 1, 2	−	0	66 ± 6	66 ± 7	930 ± 54	1000 ± 34
[16]	Retrospective	Single center	1.5T	MOLLI	Mean 3 SAx	4, 1, 2	53	0	59 ± 11	63 ± 5	891 ± 49	966 ± 27
[18]	Prospective	Multi-center	1.5T	MOLLI	Mean 3 Sax & LAx	3, 1, 2	0	0	58 ± 9	67 ± 4	961 ± 53	1029 ± 38
[24]	Prospective	Single center	1.5T	MOLLI	Mean 16 AHA	3, 1, 2	24	0	66 ± 12	66 ± 8	903 ± 14* 888 ± 70**	1001 ± 22
[20]	Prospective	Single center	1.5T	ShMOLLI	Mean mid-septum SAx & LAx	2, 1, 2	0	0	72 ± 6	−	906 ± 68	970 ± 21
[22]	Retrospective	Single center	1.5T	ShMOLLI	Mid-septum SAx	4, 1, 2	−	0	60 ± 15	66 ± 9	863 ± 23	938 ± 21
[25]	Prospective	Single center	1.5T	ShMOLLI	Mean basal-mid-septum SAx	3, 1, 2	60	0	74 ± 6* 78 ± 7**	75 ± 7	904 ± 46* 853 ± 50**	968 ± 32
[26]	Prospective	Single center	1.5T	ShMOLLI	Mean basal-mid-septum SAx	3, 1, 2	55	0	−	−	882 ± 47	968 ± 32
[23]	Retrospective	Single center	1.5T	SASHA	Mid-septum SAx	3, 1, 2	−	0	67 ± 7	62 ± 5	1053 ± 41	1180 ± 60
[27]	Prospective	Single center	1.5T	SASHA	Mean basal-mid SAx	3, 1, 2	52	0	67 ± 5	64 ± 4	1070 ± 50	1177 ± 27
[14]	Prospective	Single center	3T	MOLLI	Mean 16 AHA	4, 1, 2	14	0	67 ± 12	67 ± 2	1112 ± 49	1232 ± 12
[17]	Prospective	Single center	3T	MOLLI	Mean 16 AHA	4, 1, 2	19	0	60 ± 5	62 ± 3	1164 ± 34	1235 ± 25
[19]	Prospective	Single center	3T	MOLLI	Mid-septum SAx	4, 1, 2	21	0	59 ± 5	60 ± 4	1170 ± 37	1238 ± 18
[21]	Prospective	Single center	3T	MOLLI	Mean 3 SAx	4, 1, 2	0	0	69 ± 7	66 ± 3	1236 ± 49	1334 ± 27

Values are expressed as mean ± standard deviation or as percentage of subjects. *ROI* region of interest, *SAx* short axis, *LAx* long axis, *AHA* American Heart Association, *MOLLI* modified look-locker inversion recovery, *ShMOLLI* shortened MOLLI, *SASHA* saturation-recovery single-shot acquisition, *LVH* left ventricular hypertrophy, *LVEF* left ventricular ejection fraction. *Patients without and **patients with left ventricular hypertrophy

**Table 2:** Demographic Characteristics of the Study Population

Reference	Subjects (n)		Age (yr)		Male Gender (%)	
	AFD Patients	Control subjects	AFD patients	Control subjects	AFD patients	Control subjects
[15]	35	20	44 ± 17	46 ± 16	40	55
[16]	17	70	48 ± 18	38 ± 15	47	48
[18]	72	76	42 ± 12	49 ± 15	18	50
[24]	25	21	45 ± 15	38 ± 18	24	38
[20]	44	22	36 ± 14	34 ± 10	32	36
[22]	21	70	50 ± 17	48 ± 17	95	64
[25]	63	63	39 ± 16* 54 ± 11**	54 ± 11	43	46
[26]	44	67	39 ± 16	46	39	45
[23]	6	21	47 ± 8	41 ± 16	67	47
[27]	31	23	41 ± 12	42 ± 15	48	48
[14]	20	20	41 ± 15	41 ± 7	60	50
[17]	47	17	46 ± 14	44 ± 13	30	47
[19]	38	8	45 ± 14	40 ± 14	37	63
[21]	14	7	34 ± 12	35 ± 3	0	0

Values are expressed as mean ± standard deviation or as number (percentage) of subjects. *Patients without and **patients with left ventricular hypertrophy

### Quality assessment

Summary of the quality assessment is shown in Table 1. None of the included studies received the maximum NOS quality score. The selection and definition of AFD patients and control subjects as well as the ascertainment of mapping values was adequate in all studies.

### Overall T1 values

**Fig. 2:**
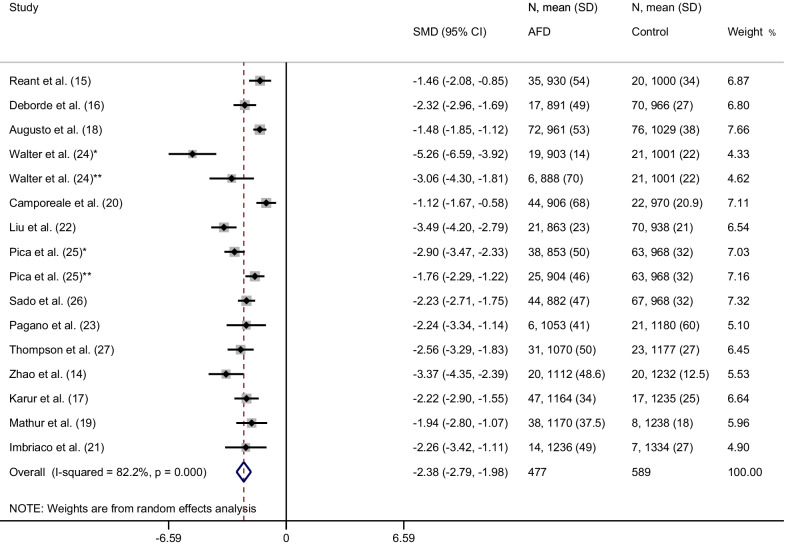
Forest plot of standardized mean difference (SMD) of native T1 values between patients with Anderson-Fabry disease (AFD) and control subjects. Native T1 values are expressed in milliseconds and reported as mean and standard deviation (SD). Squares represent individual studies with 95% confidence interval (horizontal lines). The diamond represents the pooled estimate using random-effects model. The overall intervention effect lies at the center of the diamond with right and left end points indicating the 95% confidence limits. The solid vertical line represents the reference of no increased risk, and the dashed vertical line represents the overall point estimate. *Patients without and **patients with left ventricular hypertrophy

**Fig. 3:**
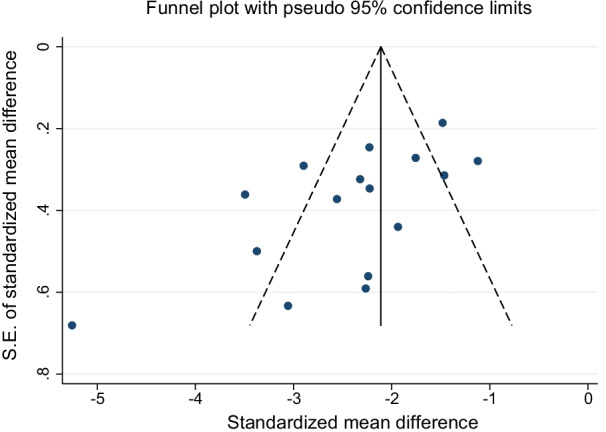
Funnel plot for standardized mean difference (SMD) of native T1 values between patients with Anderson-Fabry disease and control subjects. Each dot represents a study; the y-axis represents study precision [standard error (S.E.) of effect size] and the x-axis the effect size. Large studies appear toward the top of the graph and tend to cluster near the mean effect size. Small studies appear toward the bottom of the graph and are dispersed across a range of values since there is more sampling variation in effect size estimates. The outer dashed lines indicate the triangular region within which 95% of studies are expected to lie in the absence of biases and heterogeneity

**Fig. 4:**
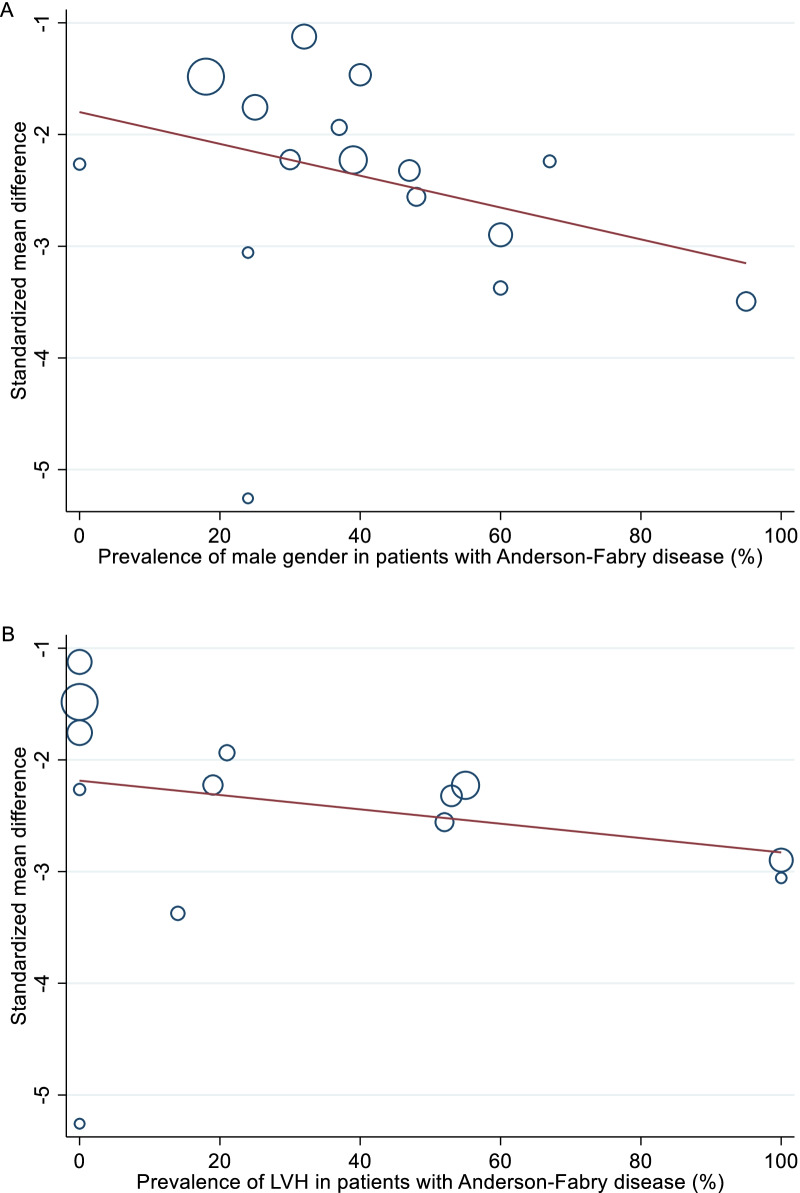
Meta-regression analysis between standardized mean difference (SMD) and percentage of male gender **A** and left ventricular hypertrophy (LVH) **B** in patients with Anderson-Fabry disease. Bubble size for each study is proportional to the inverse of the variance

The weighted mean native T1 values was 984 ± 47 ms in AFD patients and 1016 ± 26 ms in control subjects (*P* < 0.001). SMD of native T1 values between AFD patients and control subjects ranged from − 5.3 to −  1.1. The pooled SMD was − 2.4 (95% CI − 2.8–2.0) and the heterogeneity was 82.2% (Fig. 2). The funnel plot indicated publication bias (*P* = 0.01) among studies (Fig. 3). In AFD patients, an inverse correlation was detectable at meta-regression analysis between native T1 values and the prevalence of male gender (coefficient =  − 0.01, *P* = 0.002) and of LVH (coefficient =  − 0.006, *P* < 0.001) (Fig. 4).

### T1 values with 1.5T CMR scanner

#### MOLLI sequence

**Fig. 5:**
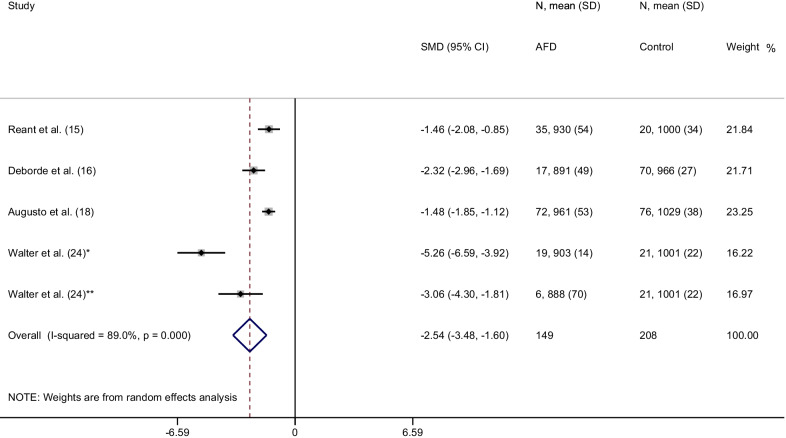
Forest plot of standardized mean difference (SMD) of native T1 values between patients with Anderson-Fabry disease and control subjects using 1.5T CMR scanner with MOLLI sequence. Native T1 values are expressed in milliseconds and reported as mean and standard deviation (SD). Squares represent individual studies with 95% confidence interval (horizontal lines). The diamond represents the pooled estimate using random-effects model. The overall intervention effect lies at the center of the diamond with right and left end points indicating the 95% confidence limits. The solid vertical line represents the reference of no increased risk, and the dashed vertical line represents the overall point estimate. *Patients without and **patients with left ventricular hypertrophy

In the 5 studies performed using MOLLI sequence in 149 AFD patients and 208 controls, the weighted mean native T1 values were 935 ± 48 ms in AFD patients and 999 ± 31 ms in controls (*P* < 0.001). SMD of native T1 values between AFD patients and controls ranged from − 5.3 to − 1.5. The pooled SMD was − 2.6 (95% CI − 3.5–1.6) (Fig. 5).

#### shMOLLI sequence

**Fig. 6:**
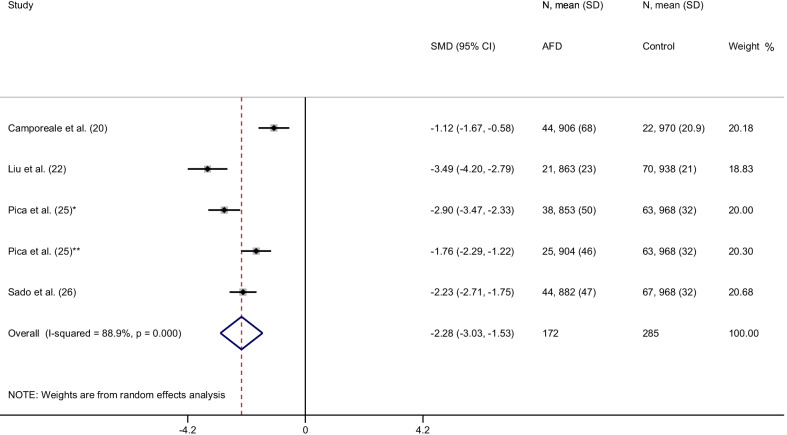
Forest plot of standardized mean difference (SMD) of native T1 values between patients with Anderson-Fabry disease and control subjects using 1.5T CMR scanner with shMOLLI sequence. Native T1 values are expressed in milliseconds and reported as mean and standard deviation (SD). Squares represent individual studies with 95% confidence interval (horizontal lines). The diamond represents the pooled estimate using random-effects model. The overall intervention effect lies at the center of the diamond with right and left end points indicating the 95% confidence limits. The solid vertical line represents the reference of no increased risk, and the dashed vertical line represents the overall point estimate. *Patients without and **patients with left ventricular hypertrophy

Five studies, accounting for 172 AFD patients and 285 controls, reported native T1 values using ShMOLLI sequence. The weighted mean native T1 values were 883 ± 50 ms in AFD patients and 961 ± 28 ms in controls (*P* < 0.0001). SMD of native T1 values between AFD patients and controls ranged from − 3.5 to − 1.1. The pooled SMD was − 2.3 (95% CI − 3.0–1.5) (Fig. 6).

#### SASHA sequence

**Fig. 7:**
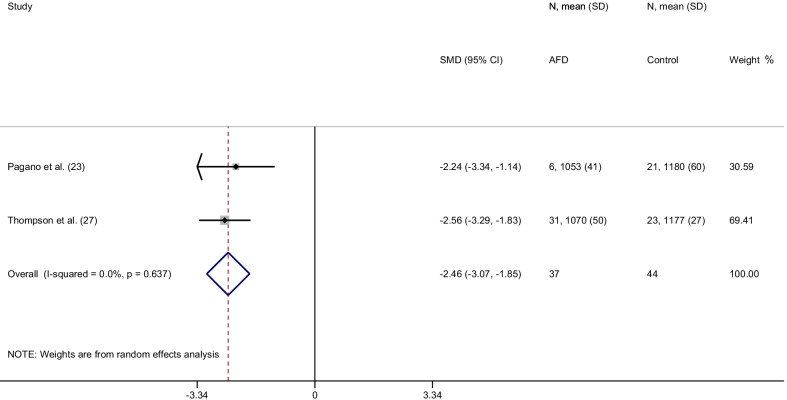
Forest plot of standardized mean difference (SMD) of native T1 values between patients with Anderson-Fabry disease and control subjects using 1.5T CMR scanner with SASHA sequence. Native T1 values are expressed in milliseconds and reported as mean and standard deviation (SD). Squares represent individual studies with 95% confidence interval (horizontal lines). The diamond represents the pooled estimate using random-effects model. The overall intervention effect lies at the center of the diamond with right and left end points indicating the 95% confidence limits. The solid vertical line represents the reference of no increased risk, and the dashed vertical line represents the overall point estimate

In the 2 studies using SASHA sequence, in 37 AFD patients and 44 controls, the weighted mean native T1 values were 1067 ± 48 ms in AFD patients and 1178 ± 43 ms in controls (*P* < 0.0001). SMD of native T1 values between AFD patients and controls ranged from − 2.6 to − 2.2. The pooled SMD was − 2.5 (95% CI − 3.1–1.9) (Fig. 7).

### T1 values with 3T CMR scanner

**Fig. 8:**
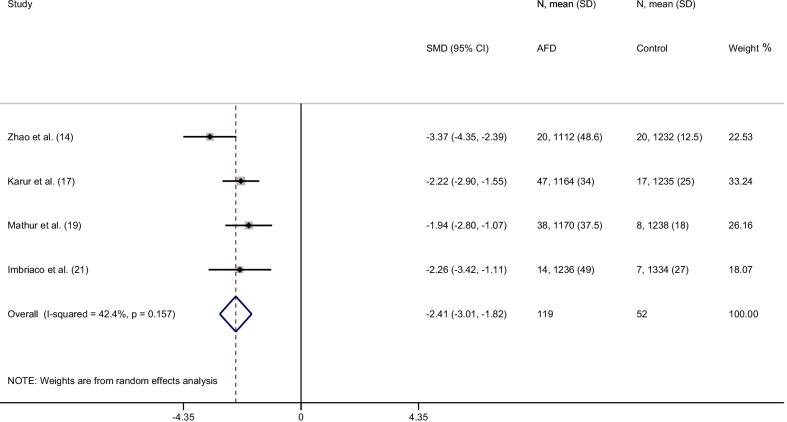
Forest plot of standardized mean difference (SMD) of native T1 values between patients with Anderson-Fabry disease and control subjects using 3T CMR scanner. Native T1 values are expressed in milliseconds and reported as mean and standard deviation (SD). Squares represent individual studies with 95% confidence interval (horizontal lines). The diamond represents the pooled estimate using random-effects model. The overall intervention effect lies at the center of the diamond with right and left end points indicating the 95% confidence limits. The solid vertical line represents the reference of no increased risk, and the dashed vertical line represents the overall point estimate

All 4 studies performed with 3T CMR scanner (including 119 AFD patients and 52 controls) used MOLLI sequence. The weighted mean native T1 values were 1166 ± 39 ms in AFD patients and 1248 ± 19 ms in controls (*P* < 0.0001). SMD of native T1 values between AFD patients and control subjects ranged from − 3.4 to  − 1.9. The pooled SMD was − 2.4 (95% CI− 3.0–1.8) (Fig. 8).

## Discussion

Quantitative analysis of myocardial native T1 values has become an important CMR tool in the detection of myocardial pathology as it can offer suitable diagnostic information about the presence of myocardial edema, lipids or iron overload, myocardial infarction and replacement fibrosis [28]. Vo et al. [29] recently evaluated the differences in myocardial T1 values between healthy subjects and patients with cardiac disorders. They performed a comprehensive meta-analysis based on several cardiac diseases, but among the 69 studies considered only one concerned AFD [26]. In our systematic review and meta-analysis, to summarize the differences in T1 values of AFD as compared to the pooled normal ranges we only included studies that measured myocardial native T1 values in AFD patients and healthy control subjects. Overall, our results confirm that native T1 mapping can be helpful in differentiating AFD patients from control subjects since overall weighted mean T1 values were lower in AFD populations than in controls.

The *primum movens* of cardiac damage in patients with AFD is the myocardial intra-cellular accumulation of glycoproteins by α-galactosidase A enzyme deficiency, with direct consequences on tissue T1 relaxation times [30]. Protein and fat have shorter T1 relaxation times by their low inherent energy and slow molecular tumbling rate, underlying the differences in T1 mapping between AFD patients and healthy control subjects [31]. Higher native T1 values with 3T scanners are justified by the T1 relaxation dependency on magnetic field strength with an increase of myocardial T1 values up to 25% as compared to 1.5T [32]. Our findings confirm T1 shortening in AFD patients compared to control subjects with different imaging equipment (1.5T and 3T CMR scanners) and different imaging protocols (e.g., MOLLI, ShMOLLI and SASHA).

Meta-regression analysis showed that heterogeneity in native T1 values among AFD patients could be explained by two major variables, gender and LVH. The cause of higher myocardial native T1 values in pre-menopausal women is not completely defined and it relies on different factors, at least in part explained by thinner myocardial walls with a predisposition to partial-volume effect, lower hematocrit values, and different myocardial characteristics due to the hormone status [33]. In addition, AFD genetic inheritance is recessive X-linked and therefore in hemizygous men, enzyme α-galactosidase A activity is completely absent, while in heterozygous women it is guaranteed by the presence of wild-type gene on the other allele [34]. Partially preserved enzymatic activity results in mild to absent intra-myocytes lipid accumulation with consequently higher values of T1 mapping compared to the male population. Thus, women carriers usually experience mild or asymptomatic form of cardiac AFD [35, 36]. However, some heterozygous female patients may manifest classical symptoms of AFD up to life-threatening expression of disease by X-chromosome inactivation (Lyonization), a process that takes place during embryo development in which randomly one of the two copies of the X chromosome is inactivated. Consequently, expression of disease in female carriers depends on the proportion of cells with inactivation of mutated or wild-type X-chromosome [37, 38].

Long-standing cardiac AFD evolves into a hypertrophic phenotype with prevalent concentric LVH based on gradual glycosphingolipid accumulation in the cardiomyocytes [39]. Accordingly, myocardial native T1 values progressively decrease as LV wall thickness increases. Advanced hypertrophic stages of cardiac AFD are characterized by myocardial inflammation and development of interstitial fibrosis, with pseudo-normalization of native T1 times and extensive myocardial LGE, characterized by a typical pattern of distribution allowing a potential differential diagnosis from other forms of hypertrophic cardiomyopathy [30, 40, 41]. Native T1 mapping represents an important biomarker, as it allows early detection of cardiac damage in a pre-hypertrophic stage and discrimination between control subjects and AFD patients without LVH. Moreover, lowering of myocardial T1 values in pre-LVH stage correlates with reduced global longitudinal strain by echocardiography and ventricular electrocardiographic abnormalities [18, 42].

Despite its clinical importance, detection of myocardial fibrosis frequently represents a late diagnostic finding, reflecting irreversible cardiac damage in a setting of LVH. Moreover, identification of myocardial fibrosis by CMR requires the administration of gadolinium, and AFD often affects the kidney, resulting in end-stage renal disease [43, 44]. Given the growing evidence showing that early administration of enzyme replacement therapy has the best chance of protecting against the effects of AFD on myocardial function and LVH, T1 mapping capability of detecting early cardiac involvement without administration of contrast agent makes it a potential standalone diagnostic tool in predicting disease progression and in timing introduction of therapy and drug monitoring [25].

## Limitations

AFD is a relatively rare disease, thus limiting the number of subjects included in the available investigations. It should be also taken into account that data regarding AFD populations investigated by 2 studies were presented separately by authors according to presence of hypertrophic pattern [24, 25]. Thus, in the present meta-analysis data were included separately for AFD patients with and without LVH. Moreover, correlations of T1 values with LGE pattern and patients’ prognosis were not assessed, due to the lack of specific data in most of the included studies.

## Conclusions

Our findings confirm the reduction of native T1 values in AFD patients compared to healthy subjects highlighting the feasibility of native T1 mapping within diagnostic work-up of AFD patients. Our results also indicate that in AFD patients the degree of T1 shortening is influenced by gender and by the presence of LVH, underlining the importance of using T1 mapping technique in the early stages of the disease before irreversible myocardial damage occurs. Although T1 mapping is useful regardless scanner field strength and imaging sequence type in proving cardiac involvement in AFD patients, there is need to standardize cut-off values according to imaging equipment and protocols to guarantee accurate and reproducible information to clinicians either for diagnostic purpose or disease management.

## Data Availability

The data generated to reach the conclusions of this meta-analysis are available from the corresponding author on reasonable request.

## Appendix

See Table 3.

**Table 3:** Literature Search Strategy*

	PubMed	Scopus	Web of science
Search	Title/abstract	Title/abstract/keyword	Title/abstract/keyword
Set	FABRY and T1 or T 1 and MAP or MAPPING or MAPS or VALUES or VALUE or RELAXATION or TIMES or TIME and cardiac magnetic resonance imaging or cardiac magnetic resonance or cardiac MRI or CMR or CMRI or cardiac or cardiac MR	FABRY and MAP or MAPPING or VALUE or TIME or RELAXATION and cardiac magnetic resonance imaging or cardiac magnetic resonance or cardiac MRI or CMR or CMRI or cardiac or cardiac MR and T1 or T 1	FABRY and TI MAPPING or MAP or VALUE or TIME or RELAXATION and CARDIAC MAGNETIC RESONANCE IMAGING or CMR or CMRI or CARDIAC or CARDIAC MR and T1 or T 1

*Capital letters indicate MeSH or Emtree terms

## Abbreviations

AFD: Anderson-Fabry disease
CMR: Cardiovascular magnetic resonance
ECV: Extracellular volume
LV: Left ventricle/left ventricular
LVEF: Left ventricle ejection fraction
LGE: Late gadolinium enhancement
LVH: Left ventricle hypertrophy
MOLLI: Modified look–locker inversion recovery
ROI: Region of interest
SASHA: Saturation-recovery single-shot acquisition
ShMOLLI: Shortened MOLLI
SMD: Standardized mean difference
